# Molecular Surveillance of Malaria Using the PF AmpliSeq Custom Assay for *Plasmodium falciparum* Parasites from Dried Blood Spot DNA Isolates from Peru

**DOI:** 10.21769/BioProtoc.4621

**Published:** 2023-03-05

**Authors:** Johanna Helena Kattenberg, Norbert J. Van Dijk, Carlos A. Fernández-Miñope, Pieter Guetens, Mathijs Mutsaers, Dionicia Gamboa, Anna Rosanas-Urgell

**Affiliations:** 1 Biomedical Sciences Department, Institute of Tropical Medicine Antwerp, Antwerp, Belgium; 2 Instituto de Medicina Tropical “Alexander von Humboldt”, Universidad Peruana Cayetano Heredia, Lima, Peru; 3 Global Health Institute, University of Antwerp, Antwerp, Belgium

**Keywords:** *Plasmodium falciparum*, Malaria, Molecular surveillance, Drug resistance, AmpliSeq custom assay, Sequencing

## Abstract

Malaria molecular surveillance has great potential to support national malaria control programs (NMCPs), informing policy for its control and elimination. Here, we present a new three-day workflow for targeted resequencing of markers in 13 resistance-associated genes, *histidine rich protein 2* and *3 (hrp2&3)*, a country (Peru)-specific 28 SNP-barcode for population genetic analysis, and apical membrane antigen 1 (*ama1*), using Illumina short-read sequencing technology. The assay applies a multiplex PCR approach to amplify all genomic regions of interest in a rapid and easily standardizable procedure and allows simultaneous amplification of a high number of targets at once, therefore having great potential for implementation into routine surveillance practice by NMCPs. The assay can be performed on routinely collected filter paper blood spots and can be easily adapted to different regions to investigate either regional trends or in-country epidemiological changes.

## Background

Surveillance of *Plasmodium* parasites has been identified by the World Health Organization (WHO) as one of the essential pillars to move towards malaria elimination in its endemic areas (WHO, 2019). Molecular parasite genotyping tools can strengthen malaria surveillance systems by monitoring the emergence and spread of drug resistance, *histidine rich protein 2* and *3 (hrp2* and *hrp3)* deletions, quantification of malaria importation risk, and characterization of changing transmission intensity. As molecular surveillance platforms transition from proof-of-concept research studies to operational incorporation into national malaria control programs (NMCPs), simple standardized laboratory protocols feasible on benchtop sequencers and automated analysis pipelines are necessary to generate reproducible results and decrease the time from sample collection to results, thereby ensuring rapid turnover of up-to-date reports for decision making and policy (WHO, 2016; MPA Committee, 2019). Various methods have been developed to investigate drug resistance markers, usually based on the amplification of loci of interest using PCR-based techniques, with amplicons detected in real time assays by amplicon sequencing (Taylor et al., 2013; Menard et al., 2016; Nsanzabana et al., 2018), or analyzed using restriction fragment length polymorphism (Eldin de Pecoulas et al., 1995; Duraisingh et al., 1998). In the past, population surveillance widely utilized genotyping tools targeting surface antigens by PCR to distinguish parasite clones (Falk et al., 2006; Koepfli et al., 2009), followed by panels of microsatellites (MS) that are not under evolutionary selection pressure and are, therefore, more suitable to inform population genetic changes (Anderson et al., 2000; Imwong et al., 2007; Karunaweera et al., 2008). More recently, genome-wide single nucleotide polymorphism (SNP) panels, capable of defining a *molecular barcode* to capture the diversity of parasite populations, have been developed and can be investigated with methods such as microarrays, real-time PCR, and deep sequencing (Daniels et al., 2008; Neafsey et al., 2008; Baniecki et al., 2015; Koepfli and Mueller, 2017; Fola et al., 2020; Tessema et al., 2022). Surveys to detect these gene deletions rely on molecular methods based on PCR assays that classify deletions based on failure to amplify targets in the *hrp2* and *hrp3* exon region (and sometimes flanking genes).

Currently, there is no multifunctional tool that includes a combination of more than two types of markers (i.e., SNP-barcodes, drug resistance, etc.) to serve several use cases. In addition, the characterization of *hrp2* and *hrp3*-deletions relies on PCR assays that classify deletions based on failure to amplify targets. The few existing tools that combine population markers with drug resistance target short regions around validated drug resistance SNPs, missing the potential to detect novel resistance-associated mutations. Furthermore, many SNP-panels were designed from genomes across the world and lack the resolution to study subtle patterns on a smaller geographical scale. Therefore, we have developed a targeted amplicon next-generation sequencing (NGS) assay for molecular surveillance of *Plasmodium falciparum* parasites that combines a specifically designed barcode for the target country, 13 full-length resistance-associated genes (Table 1), *hrp2* and *hrp3*, and an *apical membrane antigen 1 (ama1)* microhaplotype region. The novelty of the assay is its high number of targets multiplexed in one easy workflow, using AmpliSeq deep sequencing technology (Illumina, 2018), and combining phenotypic markers with a 28-SNP barcode with in-country resolution to investigate parasite gene flow in Peru. The assay uses overlapping amplicons to cover large genes. The 28 SNP-barcode was designed with in-country resolution to monitor parasite strains circulating in Peru over space and time (Kattenberg et al., 2023). These SNPs were selected from a South American *P. falciparum* genome dataset selecting SNPs with a frequency of minor alleles below 0.35 and those that were not under selective pressure, prioritizing SNPs that differentiated the Peruvian samples from other parasite populations in the dataset. Noteworthy, the seq-based tool can be performed on routinely collected filter paper blood spots and can be easily adapted to different regions to investigate either regional trends or in-country epidemiological changes using different SNP-barcodes. The technology applies a multiplex PCR approach to amplify all genomic regions of interest in a rapid and easily standardizable procedure, and allows simultaneous amplification of a high number of targets at once, therefore having great potential for implementation into routine surveillance practice by NMCPs.

**Table 1.** Targeted genes of interest for drug resistance in the PF AmpliSeq assay AMQ: amodiaquine; ART: artesunate; ATQ: atovaquone; CM: clindamycin; CQ: chloroquine; DHA: dihydroartemisinin; HF: halofantrine; LF: lumefantrine; MQ: mefloquine; PG: proguanil; PPQ: piperaquine; PYR: pyrimethamine; SULF: sulfadoxine; QN: quinine. *The list of drugs to which resistance has been observed is non-exhaustive. Validated mutations are listed in the WHO report on antimalarial drug efficacy, resistance, and response: 10 years of surveillance (2010–2019).

Gene ID	Chromosome	Gene with resistance	Drug associated with resistance*
PF3D7_1218300	12	*ap2-mu*	ART; QN
PF3D7_1251200	12	*coronin*	DHA
PF3D7_0709000	7	*crt*	CQ; PPQ; AMQ
mal_mito_3	mitochondrial	*cytochrome B*	ATQ
PF3D7_0417200	4	*dhfr*	PYR; PG
PF3D7_0810800	8	*dhps*	SULF
PF3D7_1362500	13	*exonuclease*	PPQ
PF3D7_1343700	13	*k13*	ART
PF3D7_0523000	5	*mdr1*	CQ; PPQ; MQ; QN; HF; AMQ; LF
PF3D7_0112200	1	*mrp1*	ART, MQ, LF
PF3D7_1408000	14	*plasmepsin II*	PPQ
PF3D7_API04900	apicoplast	*23s rRNA*	CM
PF3D7_0104300	1	*ubp-1*	ART

## Materials and Reagents

1. AmpliSeq Library PLUS kit for Illumina (96 reactions; Illumina, catalog number: 20019102)
  Contains:
  - 1× Lib Amp mix
  - 10× Library Amp primers
  - DNA ligase
  - 5× AmpliSeq HiFi mix
  - FuPa Reagent
  - Low TE (Tris-EDTA buffer)
  - Switch solutions
2. AmpliSeq Custom DNA panel for Illumina (Illumina, catalog number: 20020495, custom design IAD179763_241; see Supplementary manifest file and APPENDIX 1 for oligo sequences)
  Contains:
  - Primer pool 1 (red cap)
  - Primer pool 2 (blue cap)
3. AmpliSeq CD Indexes Set A for Illumina (96 indexes, 96 samples; Illumina, catalog number: 20019105)
*Note: Optionally, you can also use Index Set B, C, or D.*
4. Absolute ethanol (EtOH) (Sigma-Aldrich, Merck, catalog number: 1.00983.1000)
5. Agencourt AMPure XP beads (Beckman Coulter, catalog number: A63881)
6. 96-well PCR plate, 0.2 mL (Greiner Bio-One, catalog number: 652201)
7. 8-well PCR strips (Greiner Bio-One, catalog number: 673210)
8. Strip caps for PCR pates (Greiner Bio-One, catalog number: 373250)
9. Adhesive seals for PCR plates (Westburg Life Science, catalog number: WB2-3800)
10. 1.5 mL DNA LoBind^®^ Tubes (Eppendorf, catalog number: 022431021)
11. Nuclease-free water (Lonza, catalog number: BE51200)
12. KAPA Library Quantification kit (KAPA Biosystems, Roche, catalog number: 07960298001)
  - DNA standards 1–6 (80 µL each)
  - Primer mix (1 mL)
  - KAPA SYBR^®^ FAST qPCR Master mix (5 mL)
13. Qubit^®^ dsDNA HS Assay kits (Invitrogen, Thermo Fischer Scientific, catalog number: Q32851)
  - Qubit^TM^ dsDNA HS reagent (250 µL)
  - Qubit^TM^ dsDNA HS buffer (50 mL)
  - Qubit^TM^ dsDNA HS standard #1 (1 mL)
  - Qubit^TM^ dsDNA HS standard #2 (1 mL)
14. Thin-wall, clear, 0.5 mL PCR tubes (e.g., Qubit^®^ assay tubes, catalog number: Q32856)
15. Pipette filter tips, 10 µL (e.g., Greiner Bio-One, catalog number: 771353; Biofil, catalog number: PPT150010)
16. Combitips 0.2 and 0.5 mL (Eppendorf, catalog numbers: 0030089774 and 0030089421)
17. Miseq Reagent kit v3 (600 cycle) (Illumina, catalog number: MS-102-3003)
  - Reagent cartridge
  - HT1 (hybridization buffer, 5 mL)
  - PR2 (incorporation buffer, 500 mL)
  - Flow cell
18. 1 N sodium hydroxide (NaOH) (Sigma-Aldrich, catalog number: 22146-5)
19. Tris-hydrochloride (HCl), pH 8.0(Sigma-Aldrich, catalog number: T3038-1L) , adjusted to pH 7.0
20. Tween^®^ 20 (Sigma-Aldrich, catalog number: 022431021)
21. PhiX Control v3 (Illumina, catalog number: FC-110-3001)
22. Optional to include as controls and take along in the library preparation:
  - DNA extracted from laboratory strain(s) of *P. falciparum* (e.g., 3D7 or Dd2) as positive control
    *Note: Laboratory strains can be obtained from BEI Resources, NIAID, NIH (**https://www.beiresources.org/*): P. falciparum, *strain 3D7, MRA-102, contributed by Daniel J. Carucci;* P. falciparum, *strain Dd2, MRA-150, contributed by David Walliker;* P. falciparum, *strain Dd2_R539T, MRA-1255 and* P. falciparum, *strain CamWT_C580Y, MRA-1251, contributed by David A. Fidock; and P*. falciparum, *strain IPC 4912, MRA-1241, contributed by Didier Ménard. Genomic DNA of these strains is also available from BEI Resources.*
  - DNA extracted from uninfected human blood as negative control

## Equipment

1. Multichannel pipettes 10 µL, 0.1 mL and 0.3 mL
2. Electronic multi-dispenser pipette (Eppendorf, Multipette © E3x, catalog number: 4987000029)
3. Centrifuge (Sigma Laborzentrifugen GmbH, Sigma 4-16KS, catalog number: 150507)
4. Vortex (Scientific Industries, Vortex Genie 2, catalog number: SI-0236)
5. 96-well magnet stand (Invitrogen, ThermoFisher Scientific, Dynamag^TM^-96 Side Skirted, #12027; RNA Life Technologies, Ambion^®^ Magnetic Stand-96, catalog number: 1307065)
6. Qubit 2.0 (or higher) fluorometer (Invitrogen, Thermo Fischer Scientific, catalog number: Q32866)
7. Conventional cycler (Biometra, Westburg, Professional Thermocycler Basic 96 × 0.2 mL, catalog number: 846-070-701)
8. LightCycler^®^ 480 real-time PCR system (Roche, catalog number: 04640268001)
9. MiSeq^TM^ system (Illumina, catalog number: SY-410-1003)

## Software

1. Illumina experiment Manager (Illumina)
2. Microsoft Excel (Microsoft)
3. In-house Linux-based pipeline
  - Ubuntu (Canonical, ubuntu.com)
  - Trimmomatic (Bolger et al., 2014, https://doi.org/10.1093/bioinformatics/btu170)
  - Burrows Wheeler Aligner (Li et al., 2009, https://doi.org/10.1093/bioinformatics/btp324)
  - Picard tools (Broad Institute, https://broadinstitute.github.io/picard/)
  - Genome Analysis Toolkit (GATK) (Broad Institute, https://gatk.broadinstitute.org/)
  - SnpEff (Cingolani et al., 2012, https://doi.org/10.4161/fly.19695)
4. On-Off instrument alignment
  - Local Run Manager (Illumina)
  - DNA Amplicon Module (Illumina)

## Procedure

**Figure 1.**
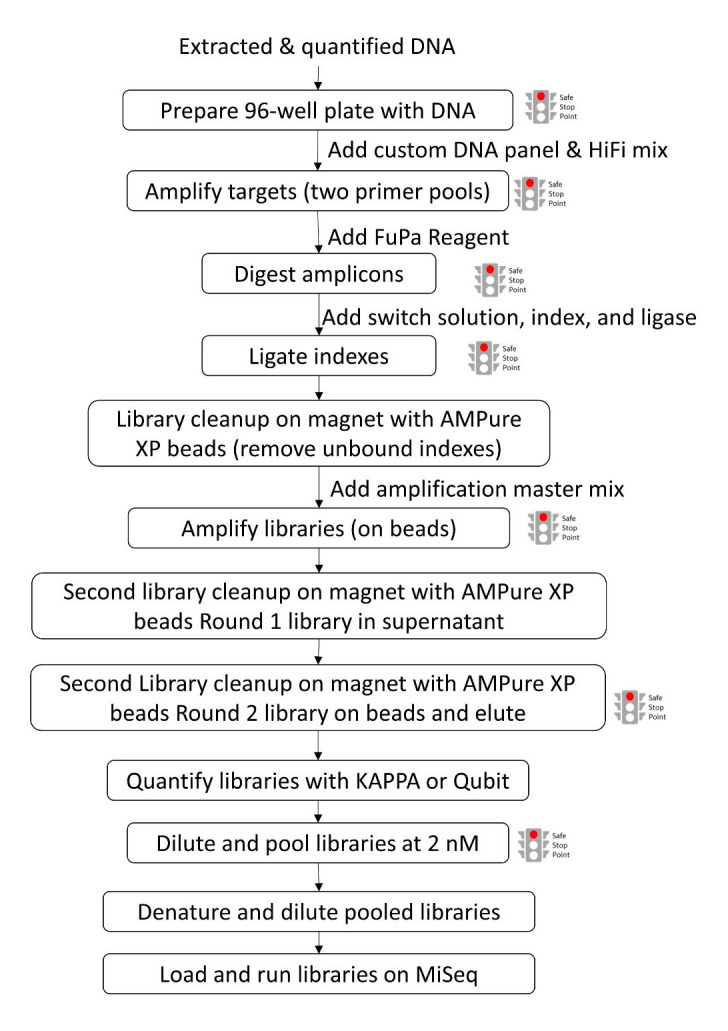
Schematic flowchart of library preparation procedures

In this protocol, target regions of parasite DNA are amplified in two reactions, followed by a library preparation where indexes are added and subsequently libraries are amplified and cleaned, quantified, pooled, and sequenced on a MiSeq system (Figure 1).

- ***P. falciparum–*infected human blood sample preparation**
  This section quantifies and prepares the DNA extracted from dried blood spots (DBS) for library preparation.
  1. Isolate DNA from DBS according to standardized protocols (e.g., DNA extractions using the Qiagen 96 DNA blood kit or E.Z.N.A. DNA mini kit).
*Note: DNA extracted from whole blood or white blood cell–depleted blood can also be used as input material. However, take note of the DNA concentrations as described in step A3.*
  2. Quantify 3–10 µL of DNA of (a subset of) samples using the Qubit^®^ dsDNA HS Assay kits, according to manufacturer’s protocol.
    - Set up the required number of 0.5 mL tubes for standards and samples. The Qubit^®^ dsDNA HS Assay requires two standards.
    - Prepare the Qubit^®^ working solution by diluting the Qubit^®^ dsDNA HS reagent 1:200 in Qubit^®^ dsDNA HS buffer. Use a clean plastic tube each time you prepare Qubit^®^ working solution. For 96 samples, prepare 20 mL working solution by mixing:
      i) 19.9 mL Qubit^®^ dsDNA HS buffer.
      ii) 100 µL Qubit^®^ dsDNA HS reagent.
    - Prepare the standard tubes:
      i) Add 190 μL of Qubit working solution to each of the two tubes used for standards.
      ii) Add 10 μL of each Qubit^®^ standard to the appropriate tube, then mix by vortexing 2–3 s. Be careful not to create bubbles.
    - Prepare the sample tubes:
      i) Add 200 μL minus the sample volume (see next step) of Qubit working solution to each of the tubes used for the libraries.
      ii) Add 3–10 μL of each DNA sample to the appropriate tube, then mix by vortexing 2–3 s. Be careful not to create bubbles.
*Note: If you suspect the sample concentration is too low, you can use a higher volume (10 μL) of sample in the Qubit reaction.*
    - Allow all tubes to incubate at room temperature (RT) for 2 min.
    - On the home screen of the Qubit^®^ 2.0 fluorometer, press DNA, then select dsDNA High Sensitivity as the assay type. The Standards screen is displayed.
    - On the Standards screen, press Yes to read the standards.
    - Insert the tube containing standard #1 into the sample chamber, close the lid, then press Read. When the reading is complete (~3 s), remove standard #1.
    - Insert the tube containing standard #2 into the sample chamber, close the lid, then press Read. When the reading is complete, remove standard #2. When the calibration is complete, the instrument displays the Sample screen.
    - Insert a sample tube into the sample chamber, close the lid, then press Read. When the reading is complete (~3 s), remove the sample tube.
    - The instrument displays the results on the Sample screen. The value displayed corresponds to the concentration after your sample was diluted into the assay tube. To find the concentration of your original sample, you can record this value and perform the calculation later in the Excel template. To calculate the concentration of your sample, use the following equation:
      Concentration = measured value (concentration of diluted sample) × (200/V), where V is the volume of sample that you started the reaction in step A2 d.ii.
    - Repeat steps j-k until all samples have been read.
  3. Recommended input concentration from the library preparation kit is 10 ng of high-quality DNA per reaction with one of the two primer pools. (The kit supports 1–100 ng DNA input. Please note, however, that due to the nature of our samples, we have a mixture of human and parasite DNA.)
    Dilute samples with too high DNA concentration (>100 ng) in low TE (supplied with AmpliSeq Library PLUS kit) to an input concentration of ~1–10 ng/μL.
*Note: In our procedures, mean DNA concentration after DNA extraction from DBS was 6.1 ± 0.3 ng/μL and we used 7.5 μL of undiluted sample (~23 ng total input per reaction) in the library preparation reactions. It is important not to exceed the 100 ng DNA input as this will negatively affect the quality of the sequencing result. For DBS samples, in our experience, the DNA concentration was always below the upper limit. After testing the DNA concentration of a few samples with varying parasite densities at the start of a study, we do not routinely check the concentration every time we do a plate to save time and costs.*
    *It is important to consider that for* P. falciparum *DBS samples, we have a mixture of human and parasite DNA. To ensure you have sufficient high-quality parasite DNA, it is important to quantify your parasite DNA in advance with a qPCR. From our validation procedure (see notes at the end of the document) we recommend including samples with* P. falciparum *densities ≥60 parasites/µl as determined by Mangold PCR (Mangold et al., 2005). This limit depends also on the amount of blood spotted on filter paper and the amount of filter paper (e.g., two pieces of ~0.5 cm^2 ^as in our case), which can impact the amount of template DNA in your sample.*
  4. Prepare a 96-well layout with the sample IDs (see Excel template “Template_AmpliSeq.xlsx”).
*Note: We usually do the library preparation for 96 samples at once, so we use one 96 sample AmpliSeq Library PLUS kit per time. You could increase or decrease the number of samples and kits used for one run on a MiSeq, but this will impact the depth per sample.*
  5. Add 7.5 µL of (diluted) DNA for each sample in the corresponding well of your layout.
  6. **SAFE STOPPING POINT:** Store the 96-well plate with prepared samples at 4 °C overnight or at -20 °C for longer periods if not immediately commencing with the library preparation procedures.
- **Amplify DNA targets**
  This section uses PCR to amplify the (overlapping) target regions of the DNA samples in two reactions with two different primer pools from the AmpliSeq Custom DNA panel. As a first step, the DNA is mixed with the AmpliSeq HiFi Mix (including a buffering solution and enzyme for amplification) and subsequently split over two new PCR plates. To the wells in each of the plates, one of the two pre-mixed pools of primers is added for amplification on a thermal cycler. There are two pools of primers (and not just one amplifying all targets), to allow for the overlap between amplicons spanning large genes (see Figure 2).
  **Figure 2.**
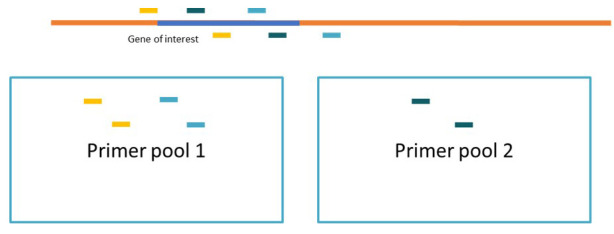
Overlapping amplicon design and primer pools. In order to allow for amplification of large genes, the custom DNA panel includes short overlapping amplicons. By using two pools of primers alternating the overlapping amplicons, we prevent amplification of the wrong primer combination in the overlapping region. After the initial two PCR reactions with the two primer pools, you add both products together and continue with a single library preparation in the next step.
  1. Thaw the following reagents:
    - 96-well plate with 7.5 µL of DNA prepared in previous section: thaw on ice if frozen.
    - 5× AmpliSeq HiFi Mix (red cap; AmpliSeq Library PLUS kit): thaw on ice and invert to mix.
    - One aliquot of 2× AmpliSeq Custom DNA panel pool 1 (red cap, AmpliSeq Custom DNA panel): thaw at RT, vortex to mix.
    - One aliquot of 2× AmpliSeq Custom DNA panel pool 2 (blue cap, AmpliSeq Custom DNA panel): thaw at RT, vortex to mix.
  2. Briefly centrifuge the 96-well plate and prepared tubes to collect all liquid at the bottom of the wells.
  3. Remove the lids/seal from the 96-well plate with DNA.
  4. To each well with sample [volume (V) = 7.5 µL], add 5 µL of 5× AmpliSeq HiFi Mix using a multi-dispenser pipette and 0.5 mL of Combitip.
  5. Centrifuge the plate briefly to collect all liquid at the bottom of the well.
  6. Mix the DNA–HiFi mixture with a multichannel pipette and transfer 5 µL to the corresponding column of a new PCR plate (labelled pool 1) and 5 µL to a second new PCR plate (labelled pool 2).
  7. To the first new plate (pool 1), add 5 µL of 2× AmpliSeq Custom DNA pool 1 (red cap) to each well using a multi-dispenser pipette and 0.5 mL Combitip.
  8. To the second new plate (pool 2), add 5 µL of 2× AmpliSeq Custom DNA pool 2 (blue cap) to each well using a multi-dispenser pipette and 0.5 mL Combitip.
  9. Seal both plates with an adhesive seal or lids and briefly centrifuge to collect all liquid at the bottom of the well. Check for air bubbles, remove by tapping/flicking the side of the well with your fingers, and centrifuge again.
  10. Place the 96-well plate in the thermocycler, set the volume to 10 µL (if applicable), and run the AMP_DNA program as in Table 2 (with heated lid on at 105 °C):
    **Table 2.** PCR cycling conditions (AMP_DNA)

Cycles	Temperature	Time
1×	99 °C	2 min
21×	99 °C 60 °C	15 s 8 min
Hold	10 °C	up to 24 h
  11. **SAFE STOPPING POINT**: If you are stopping, leave the plate on the thermal cycler at 10 °C for up to 24 h. For longer durations, store at -25 °C to -15 °C.
- **Partially digest amplicons**
  This section uses the FuPa Reagent to digest primer dimers and partially digest amplicons.
  1. From this section forward, work in a clean hood that you can radiate with ultraviolet or clean to denature DNA.
  2. Thaw the following reagents:
    - 96-well plate with amplified pool 1 prepared in previous section: thaw on ice and vortex.
    - 96-well plate with amplified pool 2 prepared in previous section: thaw on ice and vortex.
    - FuPa Reagent (brown cap; AmpliSeq Library PLUS kit): thaw on ice.
  3. If continuing with the next section (D) immediately after this section:
    Switch solution (yellow cap; AmpliSeq Library PLUS kit): thaw at RT.
  4. Briefly centrifuge the 96-well plates with amplified pool 1 and pool 2 and prepared tubes to collect all liquid at the bottom of the wells.
  5. Remove the seals from the 96-well plates with amplified pool 1 and pool 2.
  6. For each sample, use a multichannel pipette to combine the 10 µL reaction from amplified pool 2 in the corresponding well of amplified pool 1. This will result in a total volume per sample of 20 µL.
  7. Aliquot 30 µL of FuPa reagent in each well of a clean 8-well PCR strip. From here, using a multichannel pipette, transfer 2 µL of FuPa reagent to each well of the combined pool 1 + 2 product and mix. Discard the tips before continuing with the next column.
    *Note: The reagent is very viscous and due to the low volume, it is very difficult to do this with a multi-dispenser pipette and Combitip. So, use a multichannel pipette with 10 µL tips for more accurate pipetting*.
  8. Seal the plate with an adhesive seal, vortex briefly, and briefly centrifuge to collect all liquid at the bottom of the well.
  9. Place the 96-well plate in the thermocycler, set the volume to 22 µL (if applicable), and run the FUPA program as in Table 3 (with heated lid on at 105 °C):
    **Table 3.** PCR cycling conditions (FUPA)

Cycles	Temperature	Time
1	50 °C	10 min
1	55 °C	10 min
1	62 °C	20 min
Hold	10 °C	Up to 1 h
  10. **SAFE STOPPING POINT:** If you are stopping, leave the plate on the thermal cycler at 10 °C for up to 1 h. For longer durations, store at -25 °C to -15 °C.
- **Ligate indexes**
  *This section ligates Index 1 (i7) and Index 2 (i5) adapters to the fragments of each sample. The indexes are premixed in a single-use plate to ensure unique combinations. Each library must have a unique index combination for dual-index sequencing. When more than 96 samples are being included in the same sequencing run, make sure to use different index sets (four sets are available from Illumina with different combinations: Set A, Set B, set C, and/or Set D, allowing multiplexing of maximum 384 libraries). For more information see the Illumina Index Adapter Pooling Guide (**https://support-docs.illumina.com/SHARE/IndexAdapterPooling/Content/SHARE/IndexAdapterPooling/AmpliSeq/Pooling_fAS.htm*).
  To avoid library prep failure, do not combine the reagents for this section together outside the wells with the digested amplicons.
  1. Thaw the following reagents:
    - Switch solution (yellow cap; AmpliSeq Library PLUS kit): thaw at RT, vortex to mix.
    - AmpliSeq CD Index set: thaw at RT, vortex to mix.
    - DNA ligase (blue cap; AmpliSeq Library PLUS kit): thaw on ice.
  2. If continuing with the next section E immediately after this section:
    Equilibrate AMPure XP beads to RT (at least 30 min). Vortex vigorously to resuspend.
  3. Briefly centrifuge the 96-well plate with partially digested amplicons, index plate, and the prepared tubes of reagents to collect all liquid at the bottom of the wells.
  4. Remove the seals from the 96-well plate with partially digested amplicons and from the index plate.
  5. Add (**in the listed order**) to each well of the 96-well plate with partially digested amplicons:
    - 4 µL of switch solution (yellow cap) using a multi-dispenser pipette and 0.2 mL Combitip (multi-dispenser pipette set to 48 steps of 4 µL; so, you need to aspirate and dispense twice).
*Note: Alternatively, use an 8-well strip with 50 µL of switch solution aliquots and multichannel to pipette 4 µL of switch solution into each well of the 96-well plate.*
    - 2 µL of the AmpliSeq CD Index to the corresponding well of the 96-well plate with partially digested amplicons + switch solution using a multichannel pipette and 10 µL tips.
    - Aliquot 30 µL of DNA ligase reagent (blue cap) in each well of a clean 8-well PCR strip. From here, using a multichannel pipette, transfer 2 µL of DNA ligase reagent to each well of the 96-well plate with partially digested amplicons + switch solution + index.
  6. Seal the plate with an adhesive seal, vortex briefly, and briefly centrifuge to collect all liquid at the bottom of the well.
  7. Place the 96-well plate in the thermocycler, set the volume to 30 µL (if applicable), and run the LIGATE program as in Table 4 (with heated lid on at 105 °C):
    **Table 4.** PCR cycling conditions (LIGATE)

Cycles	Temperature	Time
1	22 °C	30 min
1	68 °C	5 min
1	72 °C	5 min
Hold	10 °C	up to 24 h
  8. If the index plate contains unused indexes, seal the plate and return to storage (-25 °C to -15 °C).
  9. **SAFE STOPPING POINT:** If you are stopping, leave the plate on the thermal cycler at 10 °C for up to **24 h**. For longer durations, store at -25 °C to -15 °C.
- **Clean up library**
  This section uses Agencourt AMPure XP beads to clean up the library. The library will be bound to the beads, which are carried over to the next section.
  1. Prepare the following reagents:
    - Equilibrate AMPure XP beads to RT (at least 30 min). Vortex vigorously to resuspend.
    - Freshly prepare 50 mL of 70% ethanol (EtOH) solution (mix 35 mL of 100% EtOH and 15 mL of Nuclease-free water).
  2. For the next section (F) that should immediately be continued after this section, prepare:
    - Four tubes of 1× Lib Amp mix (black cap; AmpliSeq Library PLUS kit): thaw on ice and invert to mix.
    - Two tubes of 10× Library Amp Primers (pink cap; AmpliSeq Library PLUS kit): thaw at RT and vortex to mix.
  3. Briefly centrifuge the 96-well library plate (with amplicons and index) and the prepared tubes of reagents to collect all liquid at the bottom of the wells.
  4. Remove the seals from the 96-well library plate.
  5. Add 30 µL of AMPure XP beads (vortex thoroughly before pipetting) to each well with library in the 96-well plate using a multi-dispenser pipette and 2.5 or 5 mL Combitips.
  6. Seal the plate with an adhesive seal and vortex briefly. Inspect each well to ensure the mixture is homogenous (see Figure 3); then, centrifuge briefly (low speed ~500–1,000 rpm).
    **Figure 3.**
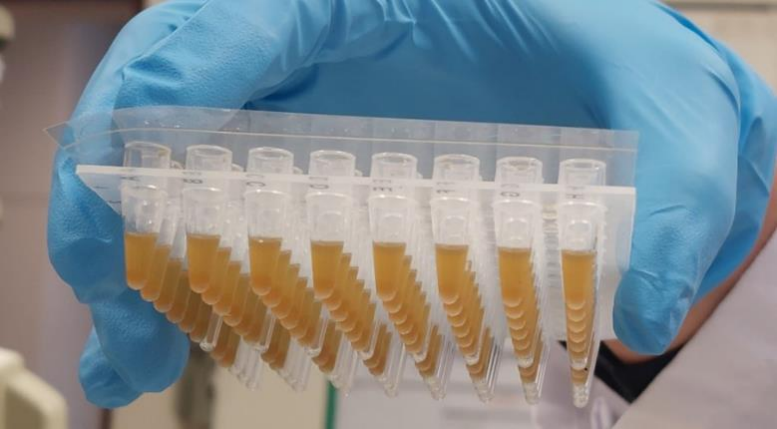
Homogenized library and beads mix
  7. Incubate the plate at RT for 5 min.
  8. Place the plate on a magnetic stand, remove the seal, and wait until the mixture is clear (at least 2 min) (see Figure 4).
    **Figure 4.**
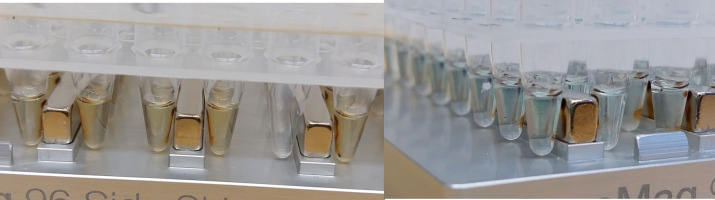
Bead mixture on magnetic stand. Left: mixture is not yet entirely clear and still a bit yellowish. Right: Mixture is clear, and the blue color of indexes added in the previous step can be seen.
  9. While on the magnetic stand, the library is bound to the beads:
    - Use a multichannel pipette (200 or 300 µL tips) to remove and discard the entire supernatant from each well.
    - Wash twice:
      i) Add 150 µL of freshly prepared 70% EtOH to each well using a multi-dispenser pipette and 0.5 mL Combitips.
      ii) Incubate at RT until the solution is clear (> 30 s).
      iii) Without disturbing the beads, remove and discard supernatant.
  10. Seal the plate with an adhesive seal and centrifuge briefly (low speed ~500–1,000 rpm).
  11. Place the plate on a magnetic stand (make sure to place in the same orientation as in steps E8 and E9 to keep the beads on the same side of the well), remove the seal, and wait until the mixture is clear (~30 s).
  12. Remove any residual EtOH as follows:
    - Use a 10 µL multichannel pipette to remove as much residual EtOH from each well as you can.
    - Air-dry the 96-well plate on the magnetic stand without seal for at least 10 min.
    - Inspect the wells to make sure the residual EtOH has evaporated. If it remains in some wells, try to remove with 10 µL pipette and continue to air-dry until the EtOH is no longer visible (Figure 4).
*Note: Overdried or cracked beads do not negatively affect the performance of the assay. However, residual EtOH causes library preparation failing in the next section by inhibiting amplification.*
- **Amplify library**
  This section uses PCR to amplify the libraries to ensure sufficient quantity for sequencing on Illumina systems. The amplification reactions contain the beads, which are carried over from the previous section. The libraries are amplified using universal primers.
  1. In the previous section you should have already prepared the following reagents:
    - Four tubes of 1× Lib Amp mix (black cap; AmpliSeq Library PLUS kit): thaw on ice and invert to mix.
    - Two tubes of 10× Library Amp primers (pink cap; AmpliSeq Library PLUS kit): thaw at RT and vortex to mix.
  2. If continuing with the next section (G) immediately after this section:
    Equilibrate AMPure XP beads to RT (at least 30 min). Vortex vigorously to resuspend.
  3. Prepare the amplification master mix in a 15 mL falcon tube (for 96 reactions) by combining the reagents as in Table 5.
    **Table 5.** Amplification master mix recipe

Reagent	Volume (µL) for one library	Volume (µL) for 96 libraries (make for 100×)	Volume (µL) for X libraries
1× Lib Amp mix (black cap)	45	4,500	
10× Library Amp primers (pink cap)	5	500	
**Total Volume (µL)**	**50**	**5,000**
  4. Vortex the amplification master mix briefly and centrifuge.
  5. Remove the 96-well library plate (from the previous section) from the magnet.
  6. Add 50 µL of amplification master mix to each well using the multi-dispenser pipette and 5 mL Combitip.
  7. Seal the plate with an adhesive seal, vortex briefly, and briefly centrifuge to collect all liquid at the bottom of the well.
  8. Place the 96-well plate in the thermocycler, set the volume to 50 µL (if applicable), and run the AMP_7 program as in Table 6 (with heated lid on at 105 °C):
    **Table 6.** PCR cycling conditions (AMP_7)

Cycles	Temperature	Time
1	98 °C	2 min
7	98 °C 64 °C	15 s 1 min
Hold	10 °C	up to 24 h
  9. **SAFE STOPPING POINT:** If you are stopping, leave the plate on the thermal cycler at 10 °C for up to 24 h. For longer durations, store at -25 °C to -15 °C.
- **Second cleanup**
  This section performs the second cleanup with AMPure XP beads for two rounds of purification.
  First round: High molecular weight DNA is captured by the beads and discarded. The library and primers are retained in the supernatant and transferred to a fresh plate for the second round of purification.
  Second round: Libraries in the saved supernatant are captured by the beads while primers remain in the supernatant. The bead pellet is saved, and libraries are subsequently eluted from the beads.
  1. Prepare the following reagents:
    - Equilibrate AMPure XP beads to RT (at least 30 min). Vortex vigorously to resuspend.
    - Low TE (bottle; AmpliSeq Library PLUS kit): thaw at RT for 45 min, vortex to mix. Can be stored at RT.
    - Freshly prepare 50 mL of 70% EtOH solution (mix 35 mL of 100% EtOH and 15 mL of nuclease-free water).
  2. Briefly centrifuge the 96-well library plate to collect all liquid at the bottom of the wells.
  3. First round: Add 25 µL of AMPure XP beads (vortex thoroughly before pipetting) to each well with ~50 µL library in the 96-well plate using a multi-dispenser pipette and 2.5 mL Combitips.
*Note: This step adds beads to the beads already in the reaction.*
  4. Seal the plate with an adhesive seal and vortex briefly, then centrifuge briefly (low speed ~500–1,000 rpm). The beads do not need to be fully resuspended.
  5. Incubate at RT for 5 min.
  6. Place the plate on a magnetic stand, remove the seal, and wait until the mixture is clear (at least 5 min).
  7. Transfer the entire supernatant (~75 µL) to a new plate. Small amounts of bead carryover do not affect performance.
**THE SUPERNATANT CONTAINS THE DESIRED AMPLICON LIBRARY!**
  8. Second round: Add 60 µL of AMPure XP beads to each well with the transferred supernatant in the new 96-well plate using a multi-dispenser pipette and 5 mL Combitips.
  9. Seal the plate with an adhesive seal and vortex briefly; then, centrifuge briefly (low speed ~500–1,000 rpm).
  10. Incubate at RT for 5 min.
  11. Place the plate on a magnetic stand, remove the seal, and wait until the mixture is clear (at least 5 min).
  12. While on the magnetic stand, perform the following steps. This time, the library is bound to the beads.
    - Use a multichannel pipette (200 or 300 µL tips) to remove and discard the entire supernatant from each well.
    - Wash twice:
      i) Add 150 µL of freshly prepared 70% EtOH to each well using a multi-dispenser pipette and 0.5 mL Combitips.
      ii) Incubate at RT until the solution is clear (> 30 s).
      iii) Without disturbing the beads, remove and discard supernatant.
  13. Use a 10 µL multichannel pipette to remove as much residual EtOH from each well as you can.
  14. Air-dry the 96-well plate on the magnetic stand without seal for at least 5 min.
  15. Remove the plate from the magnet.
  16. Add 30 µL of low TE to each well.
  17. Seal the plate with an adhesive seal and vortex briefly. Then, centrifuge briefly (low speed ~500–1,000 rpm).
  18. To ensure the beads are well resuspended, if necessary, you can mix by pipetting.
  19. Place the plate on a magnetic stand, remove the seal, and wait until the mixture is clear (at least 5 min).
  20. Transfer 27 µL of the supernatant (containing the libraries) to a new 96-well plate.
  21. **SAFE STOPPING POINT:** If you are stopping, store at -25 °C to -15 °C (up to 30 days).
- **Quantify libraries**
  Libraries should be quantified to pool the libraries of the samples at equimolar ratios for balanced sequencing output for every sample. There are two options for quantifying your libraries: using a KAPA qPCR kit or, alternatively, using the Qubit high-sensitivity reagents and fluorometer, depending on the availability of equipment in your laboratory.
**OPTION 1: KAPA library quantification**
  Follow the manufacturer’s procedures of the Library Quantification kit for Illumina Platforms (KAPA Biosystems), which is a qPCR-based method for quantification of Illumina libraries flanked by the P5 and P7 flowcell oligo sequences.
  1. Dilute the libraries to be tested 1:1,000,000 in DNA dilution buffer [10 mM Tris-HCl, pH 8.0–8.5 (25 °C) + 0.05% Tween^®^ 20]. For accurate dilution and for keeping the volume manageable, serially dilute three times 100×. So, start with mixing 3 µL of DNA + 297 µL of dilution buffer and briefly vortex, for a first 100× dilution. Then, mix 10 µL of 100× diluted DNA + 900 µL of dilution buffer, for a second dilution to 10,000× dilution, and briefly vortex. Then, mix 10 µL of 10,000× diluted DNA + 900 µL of dilution buffer for a final dilution to 1,000,000× dilution, and briefly vortex.
  2. Ensure that all components of the KAPA Library Quantification kit are completely thawed and thoroughly mixed.
  3. If the kit is used for the first time, add the primer premix (10×) (1 mL) to the bottle of KAPA SYBR^®^ FAST qPCR Master mix (2×) (5 mL). Mix thoroughly using a vortex mixer.
  4. Prepare the required internal control dilutions (e.g., PhiX library dilution) in the same way as the samples.
  5. Each library, including the controls, is measured in triplicate (so, for 96 libraries, multiple plates with KAPA Library quantification need to be performed). Prepare PCR plate layouts and include six standards and internal control (DNA standard 0 of the standards), which are all provided by the KAPA kit, as per the instructions of the manufacturer, and non-template controls (NTC, e.g., nuclease-free water or buffer).
  6. Dispense 6 µL of the master mix [KAPA SYBR FAST qPCR Master mix (2×) + primer premix (10×)] into each well of a 96-well qPCR plate.
  7. Add 4 μL of nuclease-free water to all NTC wells.
  8. Dispense 4 μL of each DNA standard into the appropriate well/tube(s), working from the most dilute (standard 6) to the most concentrated (standard 1).
  9. Dispense 4 μL of each dilution of libraries and internal controls to be assayed.
  10. Seal the PCR plate and transfer to the qPCR instrument (Roche LightCycler^®^ 480).
  11. Perform qPCR with the cycling protocol of Table 7, selecting the Absolute Quantification option in the instrument software. Adjust run parameters (e.g., reporters, reference dyes, gain settings, etc.) as required.
    **Table 7.** qPCR cycling conditions (KAPA quantification)

Cycles	Temperature	Time
1	95 °C	5 min
35	95 °C 60 °C	30 s 45 s
Melt curve	65–95 °C
**OPTION 2: Qubit DNA concentration measurement**
  Use the Qubit^®^ dsDNA HS Assay kits to determine the concentration of double-stranded DNA in the libraries, following the manufacturers’ procedures.
  1. Set up the required number of 0.5 mL tubes for standards and samples. The Qubit^®^ dsDNA HS Assay requires two standards.
  2. Prepare the Qubit^®^ working solution by diluting the Qubit^®^ dsDNA HS reagent 1:200 in Qubit^®^ dsDNA HS buffer. Use a clean plastic tube each time you prepare Qubit^®^ working solution. For 96 samples, prepare 20 mL working solution by mixing:
    - 19.9 mL Qubit^®^ dsDNA HS buffer.
    - 100 µL Qubit^®^ dsDNA HS reagent.
  3. Add 190 μL of Qubit working solution to each of the two tubes used for standards.
  4. Add 10 μL of each Qubit^®^ standard to the appropriate tube, then mix by vortexing 2–3 s. Be careful not to create bubbles.
  5. Add 197 μL of Qubit working solution to each of the tubes used for the libraries.
  6. Add 3 μL of each sample library to the appropriate tube, then mix by vortexing 2–3 s. Be careful not to create bubbles.
*Note: If you suspect the library concentration is too low, you can use a higher volume (5–20 μL) of library in the Qubit reaction. If this is the case, also adjust the volume of Qubit working solution in step H5 to make sure the final volume after adding the library is 200 μL. Take care not to use too much library as you will need sufficient volume (up to 6 µL) to make the pool in the next section. If the library concentration is too high (i.e., beyond the linear range of the Qubit kit) after the first measurement, dilute the library (e.g., 1:2 or 1:4) and measure again to get an accurate concentration.*
  7. Allow all tubes to incubate at RT for 2 minutes.
*Note: You can add the libraries to the working solution in batches of 16–24, then incubate and measure. In the meantime, keep the unused tubes in the dark, for example by covering with aluminum foil.*
  8. On the Home screen of the Qubit^®^ 2.0 fluorometer, press DNA, then select dsDNA High Sensitivity as the assay type. The Standards screen is displayed.
  9. On the Standards screen, press Yes to read the standards.
  10. Insert the tube containing standard #1 into the sample chamber, close the lid, then press Read. When the reading is complete (~3 s), remove standard #1.
  11. Insert the tube containing standard #2 into the sample chamber, close the lid, then press Read. When the reading is complete, remove standard #2. When the calibration is complete, the instrument displays the Sample screen.
  12. Insert a sample tube into the sample chamber, close the lid, then press Read. When the reading is complete (~3 s), remove the sample tube.
  13. The instrument displays the results on the Sample screen. The value displayed corresponds to the concentration after your sample was diluted into the assay tube. To find the concentration of your original sample, you can record this value and perform the calculation later in the Excel template. To calculate the concentration of your sample, use the following equation:
    Concentration = measured value (concentration of diluted sample) × (200/V), where V is the volume of library that you added, in this case 3 µL.
  14. Repeat steps 12-13 until all samples have been read.
- **Dilute and pool libraries**
  Libraries of each sample and control will be diluted with low TE to the same concentration (2 nM) individually and then pooled at equimolar ratios for a balanced sequencing output for all samples. Library quantity will vary depending on template input amount (e.g., differing parasite densities).
  1. Thaw frozen low TE buffer (AmpliSeq library prep kit) at RT.
  2. Using the library concentrations determined with the KAPA or Qubit kit (previous section), determine the molarity of the library.
    - For measurements with the KAPA kit, use the template provided by the company and standard curve included in the PCR plate to determine the size-adjusted molarity.
*Note: With KAPA kit, a library prep from a dried blood spot sample with parasite density ≥5 p/µL usually has a size-adjusted molarity within the range of 10–1000 nM.*
    - For Qubit measurements, we use the following formula: (c * 106) / (660 * library size), where c is the concentration (ng/µL) as measured with the Qubit.
*Note: You can determine the mean size of your library with, for example, the Tapestation (Agilent) or alternatively using 350 bp as the default for AmpliSeq. (With Qubit, a library preparation from a DBS sample with parasite density ≥5 p/µL usually has a size-adjusted molarity within the range of 1–100 nM.)*
    - Calculate the dilution you need to make to reach a concentration of 2 nM for each library.
  3. Dilute each library to 2 nM using low TE in a 96-well plate as indicated in the template. If the concentration (molarity) of the library (undiluted) is below 2 nM, then add 6 µL of the undiluted library to the final *2 nM* plate.
*Note: Take care not to start with a too low volume of library (at least 3 µL) for the dilution, as this will increase the inaccuracy of the final concentration. Also, take care to keep the final volume of your dilution below 200 µL; otherwise, the well of your dilution plate will overflow. If needed, dilute in a separate Eppendorf tube for larger volumes, then transfer 50 µL of the diluted library to the appropriate well in the dilution plate.*
  4. Make a pool with equal volumes of all 2 nM libraries:
    - Use a multichannel pipette to combine 5 µL from each well from the rows of the 96-well plate into one 8-well strip (i.e., make a pool from each row). Mix by pipetting 10 times.
    - Combine the entire volume of the row pools from the 8-well strip into one 1.5 mL LoBind^®^ tube.
*Note: This example for the pooling is for 96 samples. When sequencing more than 96 samples in one run, pool all 2 nM libraries of all samples to be included in the run into one final pool at equal volumes. When running more than 96 samples, take care to combine libraries prepared with different index sets; otherwise, you will not be able to demultiplex the sequences from the different plates.*
  5. Preferably, proceed to sequencing the diluted library pool soon after the dilution. If not possible, store diluted library at -20 °C up to one week.
- **Preparing files for sequencing on MiSeq**
  This section describes the steps and settings needed to prepare the sample sheet that is required to set up the sequencing reaction on the MiSeq.
  1. Using the Illumina Experiment Manager software, create a sample plate:
    - From the main screen, select Create Sample Plate.
    - Select AmpliSeq CD Indexes plate A (or B, C, or D, depending on the index kit that you used during the library prep), and then select Next.
    - In the Unique Plate Name field, enter a unique name for the sample plate. (Do not use special characters and spaces in sample IDs and plate names.) Select 2 (Dual) for the Index Reads, which should be the default setting. Select Next.
    - Select the Table or Plate tab, depending on your preferred view.
    - Enter a unique Sample ID for each well (for example by copy pasting from an Excel layout).
    - Setting the indexes (in the Table tab).
      When using the default layout from the 96-well index plates, you can auto populate the indexes: select the Apply Default Index Layout button at the bottom left.
    - When you are using a different layout than the standard index plate layout:
      i) Select a well in the Index Well field.
      ii) In the Index1 and Index2 fields, select the index adapter being used for each Index Read.
    - Check your layout; valid entries for all samples will have turned white instead of brown/grey. If everything is valid, select Finish, and then save the sample plate file in a desired location.
  2. Using the Illumina Experiment Manager software, create a sample sheet:
    - From the main screen, select Create Sample Sheet.
    - Select MiSeq and then Next.
    - Select the appropriate application (Other -> FASTQ Only, to only generate fastq files for subsequent analysis) and then select Next. (Alternatively, variants can be analyzed directly using the DNA Amplicon application and the manifest file in the basespace cloud system or local run manager.)
    - In the Reagent Kit Barcode field, enter the reagent kit ID from the label of box 1 or box 2 of the SBS kit that starts with RGT, followed by eight digits located underneath the barcode. (If unknown at this stage, you can correct this later.)
    - Select the appropriate Library Prep Workflow (AmpliSeq Library PLUS for Illumina).
    - Select the appropriate Index Adapter (AmpliSeq CD Indexes plate A, B, C, or D, or 384 when combining more than 96 samples).
    - Index Reads should be 2 (Dual) by default.
    - Enter the Experiment Name, Investigator Name, Description, and Date.
    - Enter the expected date of sequencing.
    - Select the Paired End Read Type.
    - In the Cycles Read fields, enter one more than the number of cycles (301 for the MiSeq v3 600 cycle reagent kit) for both read 1 and for read 2.
    - In the workflow specific settings, select Use Adapter Trimming (default).
    - Select Next to continue to Select Samples for a MiSeq Sample Sheet.
    - Select samples by selecting Select Plate and then navigate to the sample plate prepared in step J1.
    - Choose wells to include in the sequencing run (Select all).
    - Select Add Selected Samples.
*Note: Make sure that all the libraries included in the pool are in the sample sheet with corresponding indexes. So, if you are running more than 96 samples in a run, add all plates to the one sample sheet.*
    - Select Finish, and then save the sample sheet file (*.csv) in the desired location. Review the sample sheet in Excel (there should be no spaces or special characters in the sample IDs).
- **Final preparation for sequencing**
  In this section, the pooled library is denatured and diluted before loading on the MiSeq cartridge for sequencing. A control library (PhiX) is added to the pool at the same concentration and will act as a control for the sequencing reaction on the MiSeq. In addition, by adding PhiX, you will increase the nucleotide diversity during the sequencing run, which will increase the sequencing quality, as the *P. falciparum* genome, and hence also the targeted regions in this assay, are high in A and T nucleotides.
  1. Prepare a fresh dilution of 0.2 N NaOH:
    - Combine the reagents in an Eppendorf tube:
      i) 800 µL nuclease-free water.
      ii) 200 µL 1.0 N NaOH.
    - Mix the tube by inverting several times.
    - Use within 12 h.
  2. Prepare HT1:
    - Thaw the HT1 buffer (Miseq reagent kit v3, Illumina) at RT.
    - Store the HT1 buffer at 2–8 °C until needed, if not used immediately when thawed.
  3. Denature libraries:
    - Combine 5 µL of library pool with 5 µL of 0.2 N NaOH.
    - Vortex briefly, then centrifuge briefly.
    - Incubate at RT for 5 min.
    - Add 5 µL of 200 mM Tris-HCl, pH 7.0.
    - Add 985 µL of pre-chilled HT1 buffer to the tube of denatured pool. The result is a 10 pM denatured library. Vortex and centrifuge briefly.
    - Place the 10 pM libraries on ice until you are ready to proceed to final dilution.
  4. Dilute library pool to final loading concentration.
    Dilute with pre-chilled HT1 buffer until the final loading concentration at a final volume of 600 μL:
    18 pM for KAPA quantified libraries
    7 pM for Qubit quantified libraries
    If you are stopping, seal the tube and store at 25°C to 15°C.
  5. Spike library pool with 1%–5% PhiX spike-in to include quality controls (5% is recommended for PF AmpliSeq libraries as GC content, and therefore library diversity, is lower).
    - Dilute stock of PhiX spike-in to a concentration similar to the final library (7 or 18 pM).
      i) Take 570 µL of 7 pM library
      ii) Add 30 µL of PhiX
    - You obtained a 7 pM library pool with 5% PhiX spike-in for loading onto the reagent cartridge and the MiSeq according to the directions of the manufacturer and Reagent kit. Keep on ice until loading.
- **Preparation of the MiSeq and loading the library pool**
  1. Perform a pre-run wash of the MiSeq as per the instructions on MiSeq control software after selecting Wash.
  2. Thaw the reagent cartridge from the MiSeq V3 Reagent kit in a water bath (or sink filled with a layer of water) at RT. Do not submerge the entire cartridge, only the base. There is a maximum water level indicator line on the cartridge that you should not surpass. It will take 1–2 h before the entire cartridge is thawed.
  3. Invert the reagent cartridge 10 times to mix the reagents. Inspect that all reagents are thawed, fully mixed, and free of precipitates.
  4. Gently tap the cartridge on the bench to reduce air bubbles in the reagents.
  5. Set the reagent cartridge aside if not using immediately, store on ice or at 2–8 °C for up to six hours. For best results, proceed directly to loading the sample and setting up the run.
  6. Locate the seal that is covering the reservoir labelled Load Samples and clean the foil with a tissue/Kimwipe and pierce it with a clean 1 mL pipette tip.
  7. Add 600 µL of prepared denatured 7 pM library pool with 5% PhiX spike-in into the reservoir marked Load Samples; avoid touching the seal.
  8. Set up the run on the MiSeq System:
    - Select Sequence using the MiSeq control software.
    - Select Set up a run with the Local Run Manager and follow the steps until the Local Run Manager is opened.
    - Select Create Run and the appropriate analysis module:
      i) Select the module GenerateFASTQ to sequence and generate fastq files that can be analyzed with the Linux pipeline described in the data analysis option.
      ii) Select the module DNA Amplicon to use the manifest file to perform the variant calling on the MiSeq system.
      *Note: You will need to have this module installed on your MiSeq system in order to use it. You will also need to copy the manifest file to the MiSeq system, as well as the reference genome following the instructions of the Local Run Manager Amplicon Analysis Module Workflow Guide (**https://support.illumina.com/content/dam/illumina-support/documents/documentation/software_documentation/local-run-manager/local-run-manager-dna-amplicon-workflow-guide-1000000048047-03.pdf*).
    - Select Import sample sheet to import the sample sheet prepared in section J.
    - Check the settings, samples, and indexes and add a name for the run and a description (optional).
    - Select Save Run. Your run is now ready for sequencing.
  9. With the local run manager still open, select the run you just created; then, select Next at the bottom right.
  10. Remove the previously loaded flow cell from the MiSeq machine by releasing the clamp with the button.
  11. Carefully clean the new flow cell with a Kimwipe or ethanol wipe until the surface is clean. Place it in the MiSeq system, close the clamp, and close the lid of the flow cell compartment. The machine will check the ID and correct placement of the flow cell. Select Next.
  12. Invert the PR2 bottle to mix and open it.
  13. Open the reagent compartment door, raise the handle until it locks, and replace the wash bottle with the PR2 bottle. Empty the waste reservoir.
  14. Close the handle and the reagent compartment door. The MiSeq will check if all materials are placed correctly and read the ID of the PR2 bottle. Click Next when the check is completed.
  15. Open the reagent compartment door and the reagent chiller door and remove the wash cartridge. Insert the reagent cartridge with your loaded library into the reagent chiller, sliding it to the back.
  16. Close the reagent chiller door and the reagent compartment door. The MiSeq will check if all materials are placed correctly and read the ID of the reagent cartridge. Click Next when the check is completed.
  17. Check the parameters of the run [e.g., Read type (Paired end), read length (2 × 300 + index reads)] and click Next when correct or Edit to make changes.
  18. The system will perform a complete pre-run system check. When complete and if everything is in order, select Start Run to start the sequencing. The run with a 600-cycle MiSeq Reagent kit V3 will take ~56 h to complete.
  19. When the run is complete, the fastq files will be ready to copy to a USB and then your computer for analysis (or, if you selected to share the run on Basespace, you can access and download it in your basespace account).

## Data analysis

**Figure 5.**
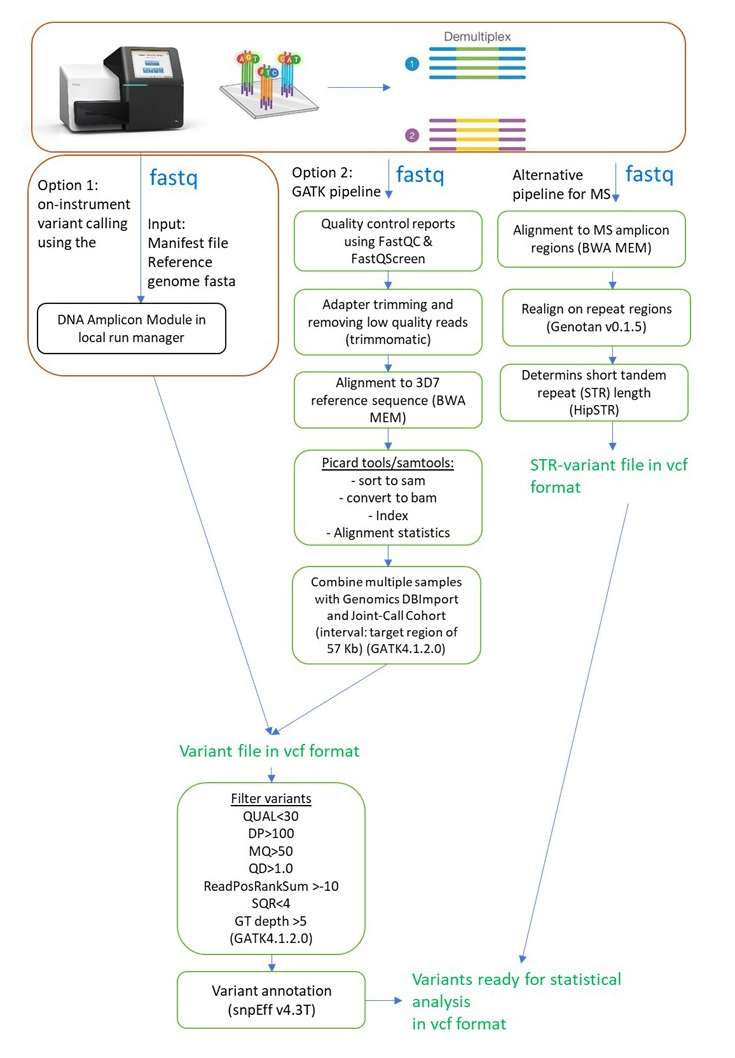
Flowchart of variant calling procedures

The Miseq sequencer generates fastq files with the raw sequencing reads that are automatically demultiplexed (i.e., using the indexes in the sample sheet generated in step J, the reads are separated for each sample and individual fastq files are generated for each individual sample). We processed these fastq files with an in-house analysis pipeline on a Unix operating system desktop computer, which is described in Kattenberg et al. (2023) and described below. Alternatively, for fast automated variant calling after sequencing, the fastq files of your run can be analyzed with the local run manager (Illumina) on the MiSeq and the DNA-Amplicon approach using the manifest file (Additional Material), according to the local run manager instructions explained in the previous section (Figure 5).

**Variant calling**

In our in-house pipeline (https://github.com/Ekattenberg/Plasmodium-AmpliSeq-Pipeline

), the resulting sequences are processed to generate a variant file (vcf), containing only the positions where the samples had a sequence that was different from the reference sequence. In the series of scripts, the following steps are performed:

1. Quality control reports are generated using FastQC. FastQ Screen was used to determine sources of contamination (e.g., human sequences).
2. Fastq files are trimmed using Trimmomatic (settings: ILLUMINACLIP: 2:30:10 LEADING:3 TRAILING:3 SLIDINGWINDOW:4:15 MINLEN:36) to remove adapter sequences and poor-quality reads.
3. Trimmed reads are subsequently aligned to the 3D7 reference genome (version plasmoDB-44) using Burrows-Wheeler aligner (v0.7.17) ( Li et al., 2009).
4. Alignment statistics are generated using Picard’s CollectAlignmentSummaryMetrics.
5. Subsequently, variants in amplicons including overlapping regions were jointly called using HaplotypeCaller (GATK, v4.1.2) and GenotypeGVCFs (GATK, v4.1.2).
6. Variants are hard filtered [QUAL > 30, overall depth (DP) > 100, RMSMappingQuality (MQ) > 50, QualByDepth (QD) > 1.0, ReadPosRankSumTest (ReadPosRankSum) > -10, StrandOddsRatio (SOR) < 4, genotype field depth (DP) > 5].
7. Finalize by annotation with SnpEff (v4.3T), resulting in 2,146 high-quality genotypes for statistical and population genetic analysis.

**Microsatellite (MS) variant-calling**

MS alleles were called using a different approach because of the short tandem repeat length.

1. The raw fastq files were aligned to reference sequences containing only the four MS amplicon regions (*poly-alpha, TA81, ARAII, and PfPK2*) using Burrows-Wheeler aligner (v0.7.17) (Li et al., 2009).
2. Subsequently, reads were realigned on repeats using Genotan v0.1.5 (Tae et al., 2014).
3. Short tandem repeat length was determined using HipSTR (Willems et al., 2017).
*Note: As HipSTR is made for diploid genomes, only the two predominant MS genotypes present in the sequencing reads are called. While this does not allow us to give exact estimates of complexity of infection (COI, i.e., number of co-infected clones), we can distinguish between single clone (COI =1) vs. multiple clone infections (COI ≥ 2, if two MS alleles are found for ≥1 MS locus).*

**Calculation of performance measures**

To measure the sequencing depth for each sample and amplicon in each sample, we calculated the median sequencing depth of all loci in the variant file for each sample or for each amplicon. To calculate the mean, we used the depth of coverage after filtering at each position in the vcf (format field DP in the vcf file). Aligned coverage is calculated as the number of bases that passed filter (from the AlignmentSummaryMetrics report) divided by the number of bases (57445 bp) targeted in the PF AmpliSeq assay.

**Inclusion criteria for analysis**

For the final analysis we include only samples with good quality data (<50% of genotype calls missing and mean aligned coverage >15) and retaining only one library in case of replicates, with the lowest proportion of genotype missingness and highest aligned coverage.

**Statistical analysis**

The presence or absence of the *hrp2* and *hrp3* genes was determined for each sample using the mean read depth of respective amplicons compared to the mean depth of all amplicons, resulting in a depth ratio. Log-transformed mean depth ratios of previously typed samples were used to define thresholds for classification for each amplicon (Kattenberg et al., 2023). A final classification of presence/absence of *hrp2* and *hrp3* was based on the proportion of amplicons with a deletion. Due to the repetitive nature and homologies of the *hrp2* and *hrp3* genes, misalignment between reads of *hrp3* with *hrp2* occurred; therefore, we used a conservative cut-off value, which sometimes resulted in a *grey zone* where deletion/presence was left inconclusive when most amplicons were not in accordance, as is explained in detail in Kattenberg et al. (2023). One amplicon for *hrp2* (AMPL3593062) was not used for the classification, as it offered no discriminatory power. A final variable for rapid diagnostic test (RDT) failure (classified as both *hrp2* and *hrp3* absent) vs. RDT detectable (*hrp2* and/or *hrp3* was present) was created, allowing also the classification of samples that were inconclusive in one of the two genes, in case the other gene was present.

Allele frequencies (AF) at barcode loci were calculated from allele depths in the vcf file to reflect true population allele frequencies in complex infections using an in-house R script.

1. First, AF was calculated for each position in each sample by calculating the ratio of the allele depths in the vcf.
2. Next, we summed the AF from the previous step for all samples, resulting in SUM-AF.
3. Then, SUM-AF calculated in the previous step was divided by the sum of within-sample allele frequencies (first step) for all alleles at that locus.

## Notes

The assay has been validated with a low error rate, high accuracy, high depth of coverage for an optimal performance to analyze blood samples collected on filter papers with *P. falciparum* parasite densities ≥60 p/µL, as determined by Mangold PCR (Mangold et al., 2005). In *P. falciparum* samples with parasite densities < 60 p/µL, selective whole-genome amplification (sWGA) prior to the PF AmpliSeq assay increases the number of reads and genotype calls, but also the error rate.

The mitochondrial targets are sequenced at a higher depth than nuclear targets, due to the higher abundance of the mitochondrial genomes in the cell. In later versions of the assay, we have removed the mitochondrial targets, which for our study purpose where less interesting. sWGA prior to library preparation balances the coverage of nuclear vs. mitochondrial amplicons.

Library quantification, in our experience, is quicker and more robust (better equalized libraries) with the Qubit kit rather than the PCR-based quantification with the KAPA kit.
